# Nanomedicine applications for the treatment of *Staphylococcus aureus* infections

**DOI:** 10.1093/femsre/fuaf068

**Published:** 2026-01-05

**Authors:** Harita Yedavally, Jan Maarten van Dijl, Anna Salvati

**Affiliations:** Department of Nanomedicine and Drug Targeting, Groningen Research Institute of Pharmacy, University of Groningen, 9713 AV Groningen, The Netherlands; University Medical Center Groningen, Department of Medical Microbiology, University of Groningen, 9713 AV Groningen, The Netherlands; Department of Nanomedicine and Drug Targeting, Groningen Research Institute of Pharmacy, University of Groningen, 9713 AV Groningen, The Netherlands

**Keywords:** *Staphylococcus aureus*, nanomedicine, intracellular infections, biofilms, antimicrobial resistance, drug delivery

## Abstract

*Staphylococcus aureus* is a Gram-positive bacterium capable of infecting multiple types of cells, organs, and tissues in the human body. Treatment can become highly challenging, especially in the case of intracellular infections and upon biofilm formation. Additionally, this pathogen has developed several antimicrobial resistance mechanisms, and resistant strains such as methicillin-resistant *S. aureus* (MRSA) are among the most difficult to treat. Within this context, nanomedicine can offer novel and more efficient treatments against *S. aureus*. Here, we first introduce the challenges in the treatment of *S. aureus* infections, focusing on intracellular infections and biofilms, and challenges associated with the development of resistance. We then provide an overview of the multiple applications of nanomedicine against *S. aureus* infection and discuss how nanomedicine may overcome the challenges in reaching this pathogen and eliminating it, including potential solutions less prone to generating resistance. Finally, we discuss the current clinical development of antimicrobial nanomedicines, where only one out of 35 completed trials has so far targeted MRSA, indicating that most research is still at the preclinical stage. Challenges in the clinical translation of antimicrobial nanomedicines are discussed, together with strategies to support the development of these promising therapeutic agents.

## Teaser

This review discusses the major challenges in treating *Staphylococcus aureus* infections and illustrates how nanomedicine can be used to address them, by reaching the places where *S. aureus* hides and by providing new antimicrobials that are less likely to generate resistance.

## Introduction and aim of the review

*Staphylococcus aureus* is a pathogen that is able to infect multiple tissues and cell types at different sites of the human body and with different extra- or intracellular outcomes (Lowy 1998, Grundmann et al. 2006, Raineri et al. 2021). In particular, the variety of intracellular infections, as well as the capacity to form biofilms on implants, together with its fast adaptation and generation of resistance mechanisms, makes *S. aureus* one of the “superbugs” that nowadays are challenging to treat and fully eradicate (Lowy 2003, Grundmann et al. 2006). Therefore, new drugs capable to fully eradicate *S. aureus* infections are constantly sought. Within this context, nanomedicine, i.e. the application of nanotechnology in health care, has attracted increasing attention in the fight against antimicrobial resistance (Duncan and Gaspar 2011, Zhu et al. 2014). In fact, because of the unique properties and cell behavior of nano-sized materials, nanomedicine can provide novel solutions to some of these problems. In particular, it offers novel methods to reach the pathogen in the places where it hides, and it provides novel modalities to treat infections.

This review will first summarize key aspects of *S. aureus* infections and describe the major challenges in treating them. Next, the review will illustrate the multiple solutions that nanomedicine can offer for addressing the different challenges presented. A particular focus will be placed on the application of nanomedicine to provide new drugs and novel therapeutic modalities, that are less likely to generate resistance mechanisms. In addition, we will illustrate how nanomedicine can be used for reaching the places where the bacteria hide and to target drugs at the organ, tissue and intracellular levels, as well as to reach and eradicate biofilms. Finally, an overview of the current clinical development status of antimicrobial nanomedicine for treatment of *S. aureus* infections will be presented, and crucial aspects that need to be addressed in order to support their development and translation will be discussed.

## Key aspects of *S. aureus* infections

**Figure 1:**
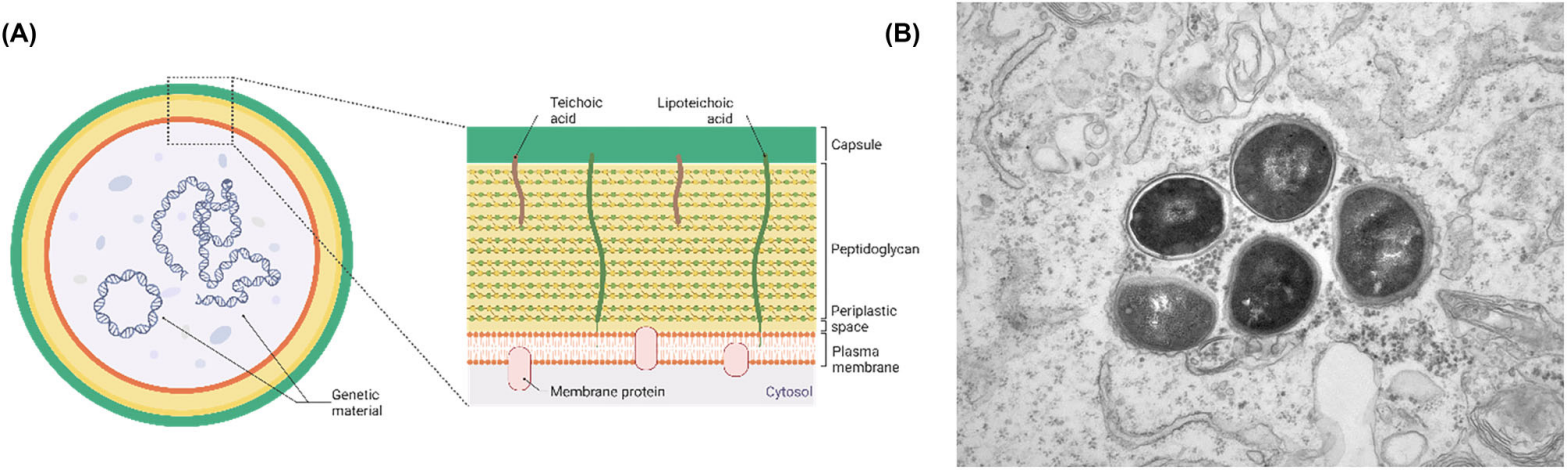
(A) Simplified representation of a *S. aureus* cell and its cell envelope. Created with BioRender.com. (B) Transmission electron microscopy image showing intracellular *S. aureus* within endothelial cells.

*Staphylococcus aureus* is a Gram-positive bacterium with a cell wall composed of a single lipid membrane surrounded primarily by peptidoglycans, teichoic acids, various proteins, and in some strains a polysaccharide capsule (Dreisbach et al. 2011, 2020, Wang et al. 2022) (Fig. 1). *Staphylococcus aureus* has a circular chromosome, which includes the core genome and a range of mobile genetic elements (Howden et al. 2023). The genes relating to antimicrobial resistance and virulence, which are found on both the chromosome and extrachromosomal elements (Sibbald et al. 2006, Dreisbach et al. 2011), can be readily transferred between bacteria. This facilitates the rapid spread of resistance and virulence traits between different lineages (Lowy 1998). Importantly, many of the encoded virulence factors serve to attach the bacteria to host tissues and cells, breach host barriers and to evade the host’s immune defenses (Gresham et al. 2000, Thwaites and Gant 2011).

**Figure 2.**
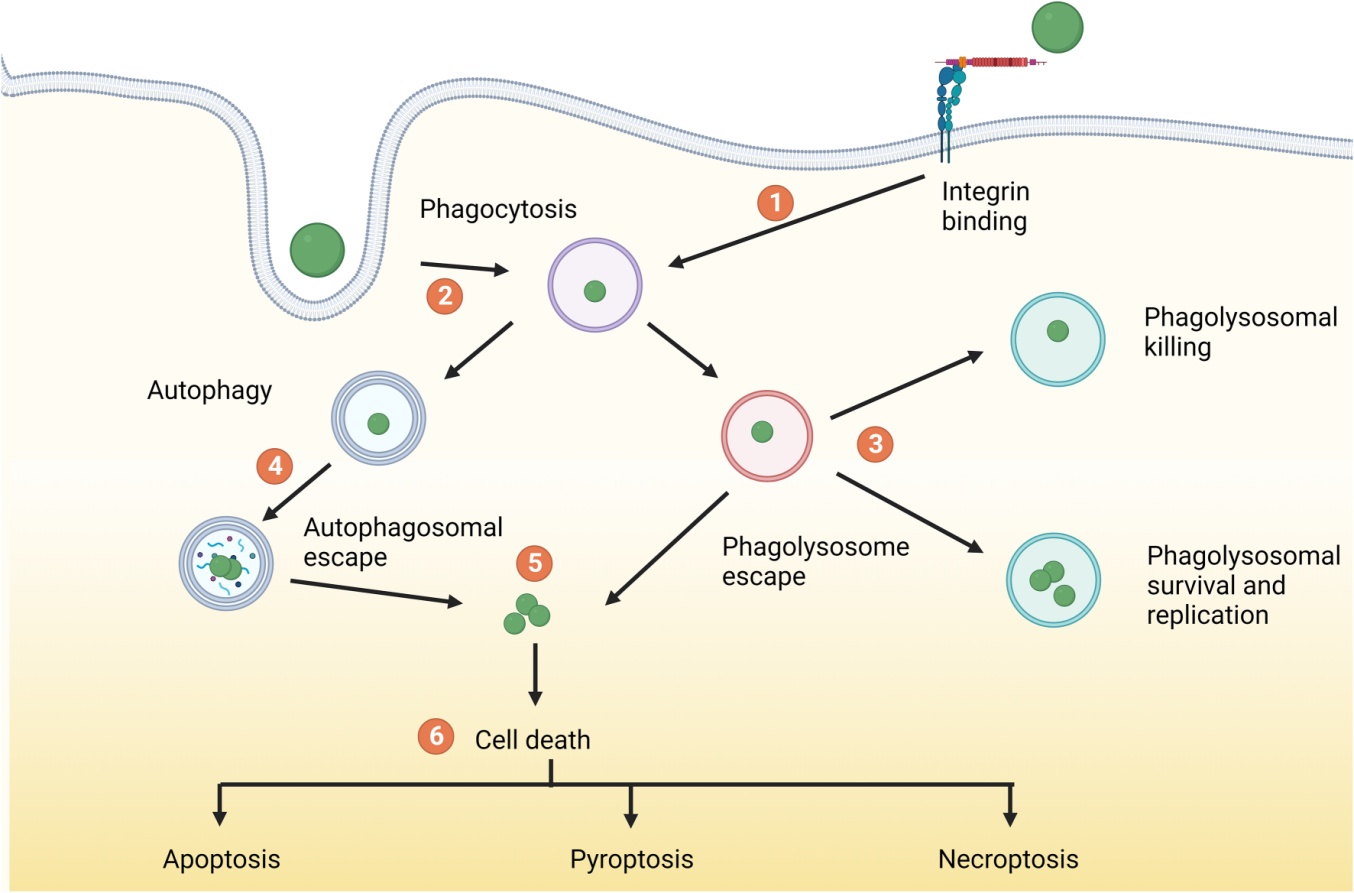
Possible intracellular fates of *S. aureus*. (1) *S aureus* can enter host cells via interaction with α5β1 integrin or (2) via phagocytosis. (3) *S. aureus* can overcome host antibacterial factors, enabling survival, and in some cases replication inside the harsh phagosome environment. (4) *S. aureus* has been associated with autophagy and can replicate inside autophagosomes. (5) *S. aureus* can escape to the cytoplasm from autophagosomes and endosomes. (6) Apoptosis, pyroptosis and necroptosis can be induced by *S. aureus*, promoting pathogen dissemination. Figure adapted from Watkins and Unnikrishnan (2020). Created with BioRender.com.

*Staphylococcus aureus* can cause a variety of diseases in a remarkably wide range of places in the human body. The broad classes of *S. aureus*-inflicted diseases are skin and soft tissue, digestive, respiratory, endovascular, and bone and joint-based infections (Raineri et al. 2021). *Staphylococcus aureus* can be present as extracellular bacteria, intracellular bacteria, and biofilm-embedded bacteria. In the bloodstream, it is usually found as an extracellular pathogen (Raineri et al. 2021). However, *S. aureus* has a high propensity to invade different cell types, including both immune cells, such as macrophages and neutrophils, and nonprofessional phagocytic cells (NPPCs), such as endothelial and epithelial cells. The intracellular environment protects the bacteria from host immune defenses, as well as from antibiotic treatment, as most antibiotics penetrate poorly into human cells (Palma Medina et al. 2019). Inside a host cell, the bacteria can have different fates (Fig. 2): they can stay enclosed in compartments along the endocytic pathway, or other membrane-confined compartments; alternatively, the internalized bacteria can escape into the host cytoplasm, where they may cause cell lysis by triggering different cell death pathways, or they can transform into a different phenotype called small colony variants (SCVs) (Tuchscherr et al. 2020). The different bacterial fates inside cells strongly depend on the bacterial strain as well as the infected host cells (Garzoni and Kelley 2009, Garciarena et al. 2015, Horn et al. 2018).

In addition to extra- and intra-cellular infections, *S. aureus* can also form biofilms in the host. Biofilms are the primary reason why implants, such as catheters, stents, pacemakers, hip and knee prostheses, and osteosynthesis materials are vulnerable to *S. aureus* colonization and infection (Götz 2002). Biofilm formation is also one of the primary reasons for the conversion of acute infections into chronic ones. A dedicated review on this topic was published by Idrees et al. (2021).

### Challenges in the treatment of *S. aureus*

*Staphylococcus aureus ’s* ability to develop resistance has long been identified as a trait that makes treatment of its infections highly challenging. In particular, due to the severity of infections and the difficulties to treat them, methicillin-resistant *S. aureus* (MRSA) is on the original ‘‘ESKAPE’’ list of high-priority pathogens (*Enterococcus faecium, S. aureus, Klebsiella pneumonia, Acinetobacter baumannii, Pseudomonas aeruginosa*, and *Enterobacter species*), deemed by the World Health Organization as threatening human health and today’s healthcare systems. Accordingly, there is a pressing need to develop new antibiotics to treat infections caused by these and an ever-growing list of other multiresistant bacterial pathogens.

The major challenges encountered while treating infections caused by *S. aureus* can be summed up as relating to two issues, the first of which is the fast occurrence of antimicrobial resistance. *Staphylococcus aureus* employs several different strategies to develop resistance to existing drugs. These include—and are not limited to—efflux pumps that pump the drug out of the bacteria, enzymes that can destroy the antibiotics, modification of targets and the formation of thick biofilms with limited permeability for antimicrobial agents.

The second challenge in the treatment of *S. aureus* infections is the difficulty in reaching the pathogen at the site of infection. As discussed above, *S. aureus* is a highly versatile pathogen capable of causing several types of diseases, and it is found in several tissues and very different cell types all over the human body. However, this bacterium does tend to have preferred niches, such as the anterior nares, throat, and gut (Raineri et al. 2021). It can also behave as a facultative intracellular pathogen, hiding inside different phagocytic and nonphagocytic cells to avoid the immune responses or drug treatment, where the bacterium can develop into SCVs (Proctor et al. 2014). These are difficult to reach due to their intracellular location, and difficult to treat due to the thickening of their cell wall, slowed-down growth and division, and low metabolic activity. In addition, *S. aureus* can also form biofilms at diseased or damaged sites, which are much more difficult to treat as the extracellular polysaccharide layer secreted by the biofilm is very difficult to fully penetrate. Once a biofilm infection has been established, there are very few treatment options available, and the usual route taken is the removal of the implant along with the surrounding tissue (López-Álvarez et al. 2022, Rosman et al. 2022). For both the persisters and SCVs that accumulate inside human cells and the bacteria embedded in biofilms, it is important to bear in mind that their growth is severely reduced, whereas essentially all clinically approved antibiotics act on growing cells that are metabolically active.

Many antimicrobial drugs are commonly administered orally, and are characterized by poor bioavailability. Being small molecules, which can diffuse and partition in the body, the systemic distribution of antibiotics often leads to a low concentration at the infection site. Poor delivery can make the drug ineffective in fully eradicating the pathogen, and can also promote the emergence of novel mechanisms of resistance. Most of the drugs used today are also characterized by uneven tissue distribution and poor cell penetration, also leading to poor delivery. Thus, treatment usually involves frequent high doses, which can lead to unwanted side effects and poor patient compliance. Overall, the current treatments are far from ideal.

Indeed, given the potential impact of fully drug-resistant bacteria both in the hospital settings and within the wider community, identification, and development of new treatment strategies is of the utmost importance. Several efforts have been focused on the development of vaccines and therapeutic antibodies against *S. aureus*. However, unfortunately, at present none of the vaccine candidates have made it past clinical trials into the market (Wong et al. 2022, Howden et al. 2023).

Within this context, nanomedicine is emerging as a new field which may provide novel solutions to reach this pathogen and treat infections.

## Nanomedicines against AMR

**Figure 3.**
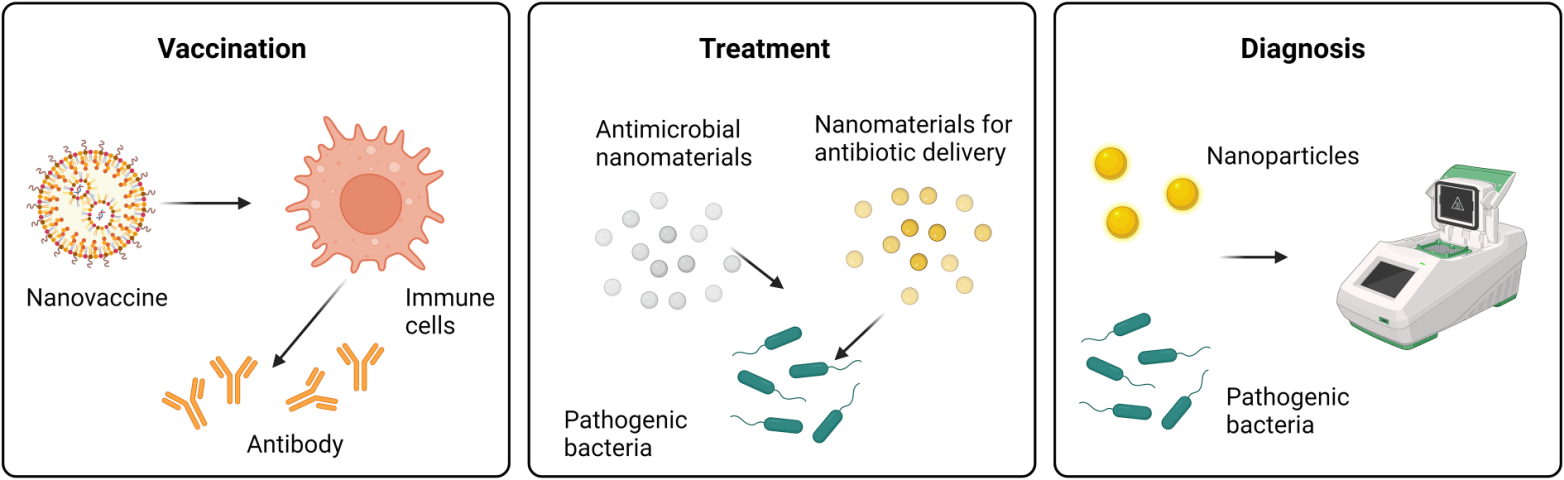
Nanomedicine applications in the management of microbial infection. Created with BioRender.com.

Nanomedicine is defined as the application of nanotechnology for healthcare. Nanomaterials are materials with at least one dimension in the size range of 1–100 nm (Duncan and Gaspar 2011). They have found several different applications in the management of infectious diseases. In addition to treating infections, they can be used in diagnosis, the development of vaccines, and in medical devices (Zhu et al. 2014) (Fig. 3).

Due to their small size and high surface area to volume ratio, nanoparticles interact with cells in a similar fashion as other nano-sized objects like viruses, proteins, and cholesterol nanoparticles. Consequently, they distribute in organisms and within cells in different ways than the small molecule drugs that are currently on the market (Salvati et al. 2011, Francia et al. 2020, Mitchell et al. 2021, Elumalai et al. 2024). In fact, nanoparticles are too large to simply diffuse into cells and, rather, they interact actively with the cell and are taken up by endocytic pathways and trafficked to specific subcellular locations (Bareford and Swaan 2007, Patel et al. 2019, Francia et al. 2020, Rennick et al. 2021). Additionally, because of their relatively large size, which limits renal clearance, they have a longer circulation time in the body. This avoids problems encountered by small molecule drugs which, instead, can passively diffuse and distribute everywhere in the body, often leading to unwanted side effects and rapid clearance by renal filtration. Thus, different nano-sized materials have been developed and are already in the clinic as drug carriers to encapsulate drugs and improve their delivery to the targeted site (Peer et al. 2007, Anselmo and Mitragotri 2016, 2021, Wolfram and Ferrari 2019). Nano-sized drug carriers can also protect drugs from enzymatic degradation, further increasing the drug circulation time. Additionally, they can be functionalized for targeted delivery thus reducing side effects, and they also allow for combinatorial therapies, which can be more efficient in eradicating ‘‘difficult’’ multidrug-resistant pathogens, such as *S. aureus* (Liew et al. 2022, Andrade et al. 2023). Thus, nanomedicine is uniquely positioned to tackle some of the specific challenges of *S. aureus* infections with respect to both reaching the pathogen and dealing with resistance (Briones et al. 2008, Gao et al. 2014, 2021, Liew et al. 2022, Zou et al. 2023).

Nanoparticles with very different properties, such as material, size, shape, hydrophobicity, charge, roughness, and elasticity (among others) can be easily designed and engineered. These properties determine how the nanoparticles interact with the body, as well as the different cell types (Blecher et al. 2011). The wide range of engineering options makes nanoparticles very interesting materials indeed. *In vivo* properties like serum protein interactions, sequestration by the immune system, blood circulation time, biodistribution, cellular recognition, and internalization are all potentially tunable, albeit to varying degrees of success. The ideal nanoparticle for drug delivery applications should display a reduced clearance, increased retention time, a biodistribution tuned by active targeting, and low cytotoxicity to the host.

Nano-sized drug carriers can be made from a variety of materials, further expanding their engineering potential. For instance, biocompatible materials like poly(lactic-co-glycolic acid) (PLGA), poly lactic acid (PLA), chitosan, gelatin, alginate, and lipids can be used. PLGA is approved for use by the Food and Drug Administration (FDA) of the USA, whereas poly ethylene glycol (PEG) can be used as a coating to increase the circulation time and, accordingly, PEG is already widely used in currently approved nanomedicines (Joralemon et al. 2010). Lipid nanocarriers have been particularly useful in the delivery of the recent mRNA-based Pfizer/BioNTech and Moderna vaccines. Liposomes are also attractive as they can encapsulate both hydrophobic and hydrophilic drugs and, accordingly, they are the most frequently applied drug carriers among the currently approved nanomedicines. Natural polymers such as alginate and chitosan can also be used and have the added advantage of allowing for encapsulation without the use of organic solvents, which need to be removed for clinical applications (Reis et al. 2006). Among the inorganic materials, gold has been explored, because of the ease of functionalization using thiol chemistry (Yeh et al. 2012), while silver nanoparticles are routinely used in wound dressings and several over-the-counter products (Marassi et al. 2018).

**Figure 4.**
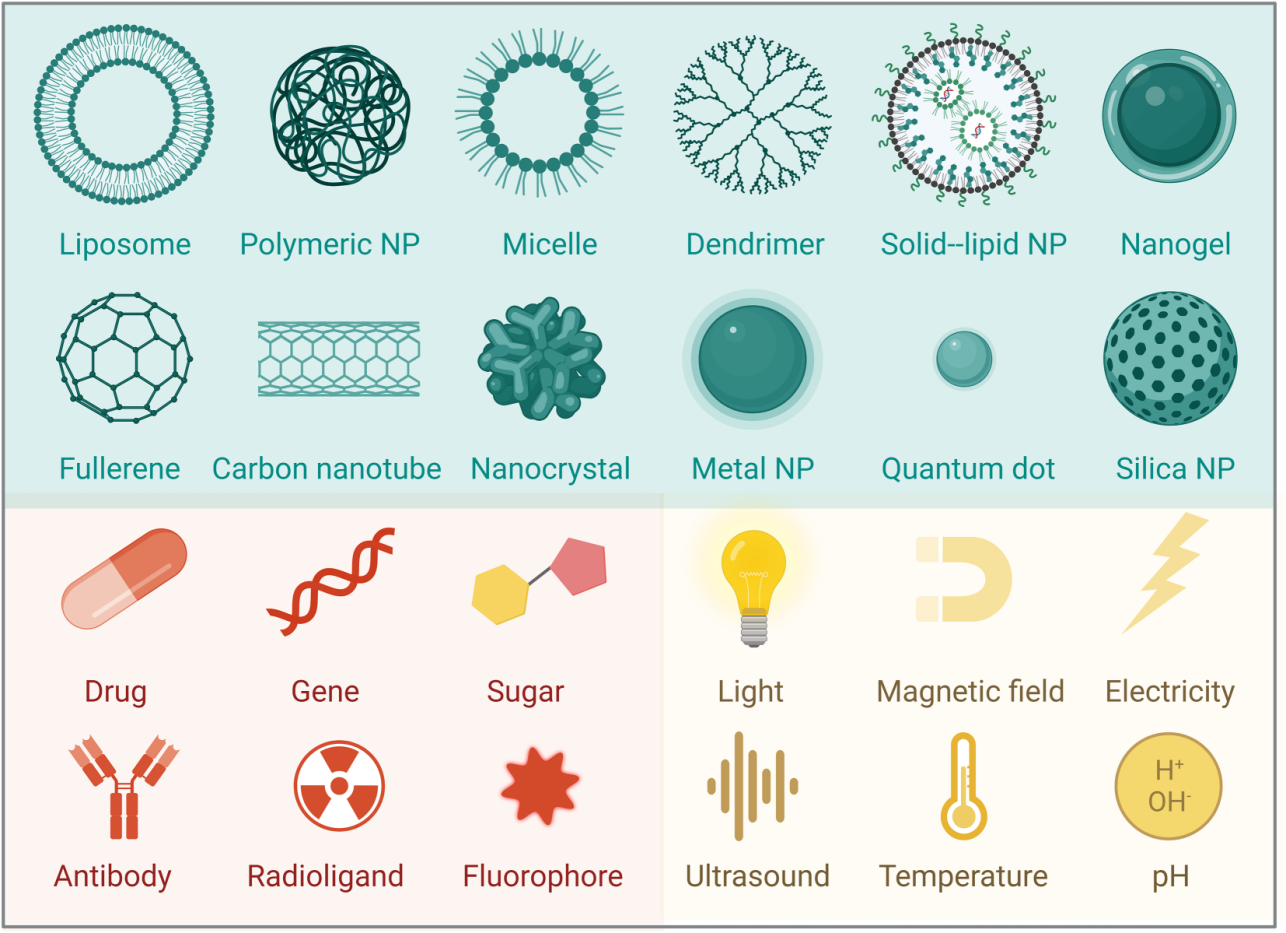
Antibacterial nano strategies. Nanoparticles can be used to deliver antibiotics. Additionally, metallic, organic, biomolecular, radio-, and antibody-modified nanoparticles can effectively destroy bacteria with multiple mechanisms and their potency can be enhanced with addition of ultrasound, magnetic field, light, and ionizing radiation properties. Created with BioRender.com.

Importantly, not only can nanomaterials be used as drug carriers for antimicrobial agents, but some nanomaterials have proven intrinsic antimicrobial activity. A variety of organic and inorganic materials fall under each type (Chakraborty et al. 2022). The class of nanomaterials with inherent antimicrobial properties includes primarily metal and metal oxide nanoparticles (Joshi and Patil 2023). Silver nanoparticles, in particular, have been known to present antimicrobial properties since ancient times, and they have a broad spectrum, showing activity against over 150 different pathogens (Marassi et al. 2018). Cationic peptides, polymeric nanoparticles, dendrimers, and carbon-based structures like nanotubes and fullerenes also can show antimicrobial properties (McNamara and Tofail 2017). Conversely, nanoparticle-based antimicrobial drug delivery systems mainly include polymeric nanoparticles and lipid-based drug carriers, such as liposomes and lipid nanoparticles, along with some metallic and inorganic particles (Fig. 4).

**Figure 5.**
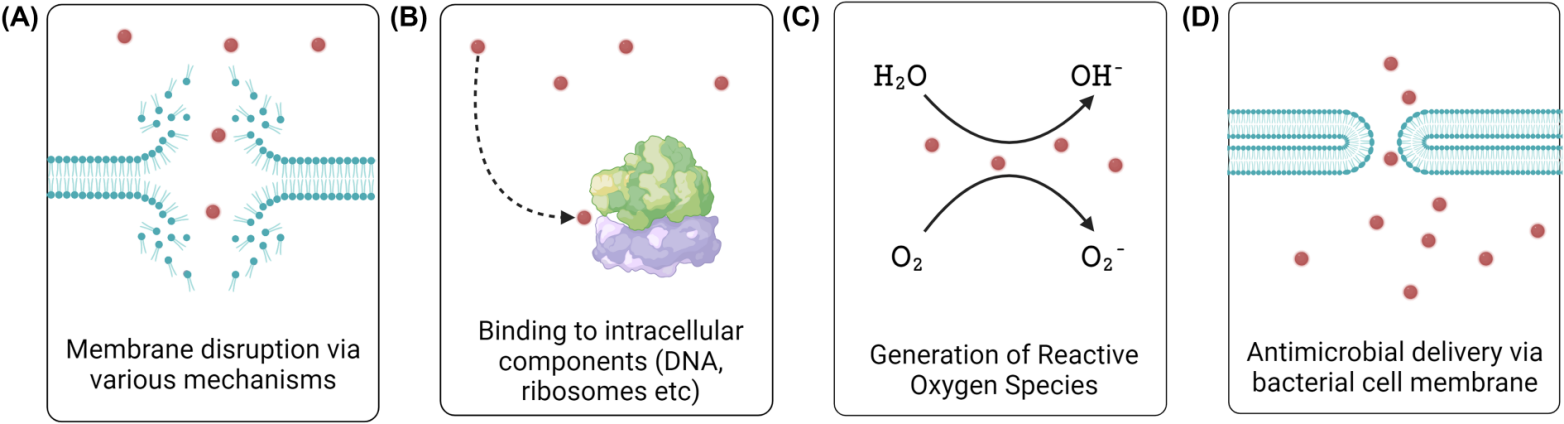
Simplified diagram showing different mechanisms by which nanoparticles can be applied to treat bacterial infections. Figure adapted from Ndayishimiye et al. (2022). Created with BioRender.com.

Nanoparticles display antimicrobial properties in several ways, which are outlined in Fig. 5. Metallic nanoparticles composed of silver (Ag), gold (Au), zinc oxide (ZnO), or copper oxide (CuO) can disrupt the bacterial membrane via different mechanisms. For instance, this can occur through electrostatic interactions, or the release of positively charged metal ions that can also perturb the bacterial membrane directly or indirectly via lipid peroxidation, but also through the generation of radicals and reactive oxygen species (ROS) (Vatansever et al. 2013). Electrostatic interactions between anionic groups on the bacterial outer surfaces and different types of positively charged nanoparticles, in particular nanoparticles with quaternary ammonium groups or other positively charged materials, such as chitosan, can also cause bacterial cell membrane damage and cytoplasmic leakage (Helander et al. 2001). Membrane perturbation has also been observed with zwitterionic surfaces. Following membrane damage, nanoparticles can access the interior of the bacteria and bind to different intracellular components, including DNA, ribosomes and proteins, but they can also disrupt or compromise the bacterial metabolism. This may be achieved by damaging DNA, inactivating enzymes, or by denaturing proteins. Nanoparticles with catalytic activities can induce and/or increase the generation of ROS, that impose oxidative stress on the bacteria. In addition, metallic nanoparticles can facilitate the release of heavy metal ions that will also lead to ROS production (Vatansever et al. 2013).

Interestingly, several nanomaterials, such as for instance gold-based nanomaterials, metal oxide nanoparticles, quantum dots, and carbon-based nanomaterials, can be employed for their capacity to interact with and adsorb light, then releasing their energy via nonradiative processes (Han and Choi 2021). Such processes lead to a local increase in temperature upon irradiation. Similar effects can be achieved with magnetic nanoparticles, such as for instance iron oxide, upon application of an alternating magnetic field (Liu et al. 2020). This kind of thermal therapies are receiving increasing attention in different fields, including for the treatment of infections, where the heat generated can be used to kill the pathogen in a targeted way in the irradiated area (Hussein-Al-Ali et al. 2014, Ibelli et al. 2018).

In addition to these intrinsic antimicrobial effects and thermal therapies, polymeric nanoparticles and lipid-based drug carriers, such as liposomes, can be used as delivery vehicles for antimicrobial agents by readily entering bacterial cell membranes. For instance, fusogenic liposomes have been employed to deliver drugs inside bacteria after fusion with the bacterial cell wall (Scriboni et al. 2019). Similarly, ultrasmall amphiphilic gold nanoparticles were shown to have a unique capacity to directly access the cytosol of mammalian cells, via energy-independent mechanisms, which might offer possibilities to target intracellularly hiding bacterial pathogens (Verma et al. 2008).

Overall, by taking advantage of the unique characteristics of nanoparticles, the use of nanotechnology in antimicrobial therapy can provide different novel strategies against bacterial resistance and to improve the delivery of antimicrobial drugs. A more detailed description of the nanotechnology solutions is presented in the following paragraphs.

### Overcoming resistance

**Figure 6.**
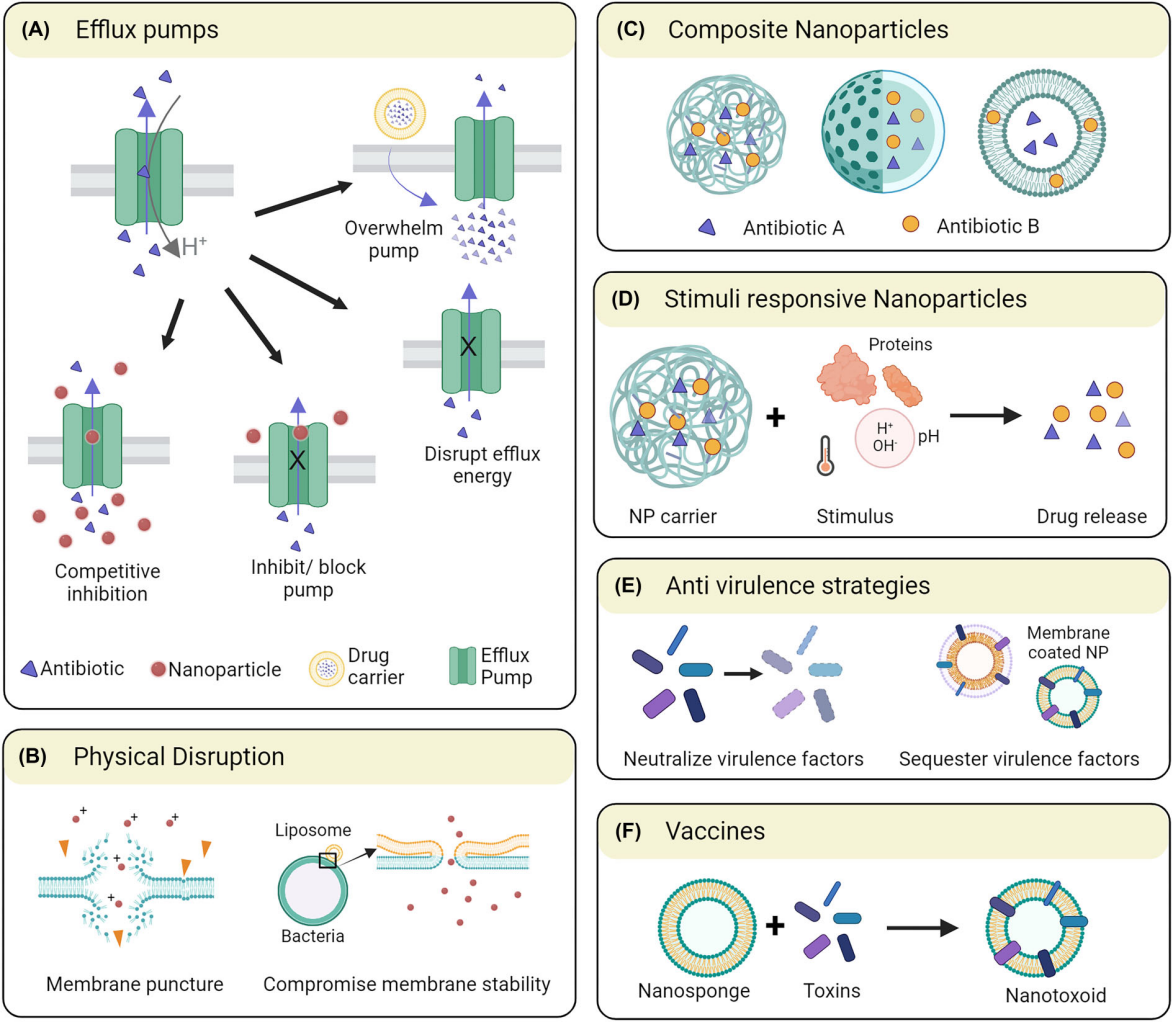
Examples of different ways nanoparticles can be designed to overcome antimicrobial resistance. These include nanoparticles that antagonize antibiotic efflux pumps (A), compromise the bacterial membranes (B), deliver multiple antibiotics simultaneously (C), respond to particular bacterial stimuli for antibiotic delivery (D), neutralize the bacterial virulence (E), or serve in the delivery of antigens for vaccination (F). Created with BioRender.com.

During their evolution bacteria have developed several ways to negate the effects of antibiotics, including inactivating the drug enzymatically, using active efflux to pump out the drug, mutating targets and preventing drug entry (Brooks and Brooks 2014, Karam et al. 2016). Nanoparticles can be designed to target these specific defenses in different ways (Fig. 6).

#### Efflux pumps

Bacteria employ membrane-embedded transporter proteins called efflux pumps to actively pump drugs out of the cell, lowering the dose available. Such pumps are encoded chromosomally or on plasmids. *Staphylococcus aureus* can express over 10 known efflux pumps (Costa et al. 2013), which confer resistance to several antibiotic classes such as macrolides, quinolones, and tetracyclines among others, as well as biocides, antiseptics, and disinfectants (e.g. quaternary ammonium compounds). Metal nanoparticles can act as efflux pump inhibitors by competing for the binding to the active site of the efflux pumps or by blocking them (Sapula and Brown 2016). They can also release metal ions which can disrupt the bacterial membrane potential, resulting in a reduction of the driving force for efflux pump activity. ZnO, Ag, or polyacrylic acid coated magnetite and Cu nanoparticles were demonstrated to display similar inhibitory effects (Mishra et al. 2022). Liposomal formulations can also be used as agents to counteract the activity of efflux pumps, since they can be loaded with drugs at high concentration that can be directly delivered to bacterial membranes by membrane fusion. In this way, they can release a high volume of the active drug molecule locally, overwhelming the efflux pumps, and allowing the delivery of a sufficient dose of the antibiotic to show a significant antimicrobial effect (Drulis-Kawa and Dorotkiewicz-Jach 2010, Gao et al. 2013, Ma et al. 2013).

#### Physical disruption

The cell wall of bacteria is generally negatively charged due to the teichoic acids present in them (Sarvas et al. 2004). Consequently, cationic peptides have been explored to target them in a similar fashion as many naturally produced cationic peptides of humans, animals and bacteria (Friedrich Carol et al. 2000). Likewise, several types of cationic nanoparticles, such as nanoparticles with quaternary ammonium groups or positively charged dendrimers and polymers, such as chitosan, have been used for triggering similar effects (Helander et al. 2001, Liu et al. 2009, Jiao et al. 2017). However, many of the latter agents do have the disadvantage of being detrimental to the negatively charged mammalian membranes as well. Lipidic formulations can also be employed to interfere with the bacterial membrane stability by integrating amphiphilic free fatty acids into the bacterial membrane at random sites. These have been effective against various Gram-negative bacterial pathogens as well as MRSA (Huang et al. 2011, Wang et al. 2011). Although, the effects of such lipidic formulations on human host cells still need to be characterized, they have been tested *in vivo* in mouse models for *Helicobacteri pylori* infection and show promise for being extended to MRSA as well (Thamphiwatana et al. 2014). Several metal nanoparticles have also a demonstrated capacity to induce physical disruption of bacterial cells, including but not limited to Ag, Cu, and ZnO nanoparticles. As pointed out above, Ag nanoparticles are known to be antimicrobial and are conventionally used in wound dressings and topical ointments. When synthesized in specific shapes like rods or prisms, they can also physically puncture the cell wall of bacteria, causing their contents to leak out. Antimicrobial photosensitizers can also be loaded into liposomes, as shown by Tsai et al. (2009), where hematoporphyrin loaded in liposomes and micelles was shown to eradicate bacteria in a murine infection model at lower concentrations than the free photosensitizer. A massive advantage of this approach is that physical nonspecific disruption of bacterial membranes directly leads to leakage of cytoplasmic contents and bacterial death. It would be very hard to develop resistance against this mechanism (Bispo et al. 2020, 2022a). Nevertheless, eventual toxicity towards the host should also be carefully examined.

#### Composite nanoparticles

Combinatorial approaches to antibiotic treatment have been employed in the clinic as a means to fight resistant bacteria with higher efficacy (Bayramov and Neff 2017). However, different drugs have different pharmacokinetic profiles and physicochemical properties. Coordinating the administration of different drugs to allow concurrent antimicrobial activity can pose challenges. Nanoparticle drug carriers can be used here as a solution to this problem, and they have already been well-studied for the co-encapsulation of drugs with varying physical properties in terms of size, charge and hydrophobicity. Liposomes and polymeric nanoparticles are the most explored in this regard. The resulting nanomedicines carry multiple types of drugs within the same drug carrier and, in this way, they allow uniform tissue penetration and distribution of all component drugs, promoting additive or even synergistic effects (Gao et al. 2014). Some examples of these include: daptomycin–clarithromycin liposomes, vancomycin–cefazolin liposomes, and oleic acid-gentamicin liposomes (Rukavina et al. 2018).

Another popularly employed approach is the use of metallic nanoparticles conjugated with antibiotic molecules in order to block efflux pumps as mentioned in the previous section, thus increasing antibiotic efficacy (Dey et al. 2022). For instance, Banoee et al. (2010) used zinc oxide particles complexed with ciprofloxacin against *S. aureus*, where ZnO blocked the efflux pump, allowing ciprofloxacin to act on the bacteria. A similar effect was observed for ampicillin-Au nanoparticles, where the efflux pump was blocked allowing the antibiotic to show effect (Brown et al. 2012). Ag nanoparticles have been combined with blue light, also known to possess antimicrobial activity due to photothermal effects, while delivering antibiotics, such as amoxicillin, rifampicin, azithromycin, clarithromycin, cefotaxime, linezolid, and other antimicrobials such as cephradine, vildagliptin, and simvastatin. The resulting combined triple activity of the silver nanoparticles, blue light irradiation and the delivered antibiotics resulted in increased efficacy against MRSA (Akram et al. 2016). In another study, gold nanoparticles combined with gentamicin provided continuous drug release over days, with additional antimicrobial effects triggered by the gold. Similarly, daptomycin-loaded gold nanocages conjugated to antibodies to target two different *S. aureus* lipoproteins effectively killed MRSA in biofilms (Meeker et al. 2018).

Several other combinatorial approaches have been employed as well. Combinations of porphyrin-type photosensitizers with polycationic liposomes enhanced their antibacterial effect, as the liposomes disorganized the bacterial wall, thereby enhancing photosensitizer permeability (Ferro et al. 2007). As for organic nanoparticles, ceftriaxone-loaded chitosan nanoparticles were assessed in a neutropenic mouse model with reasonably good results, showing a 41% decrease in MRSA. This however is not sufficient for antimicrobial therapy and further optimization is necessary (Mushtaq et al. 2017). More recently, in another interesting application, red blood cell coated nanoparticles consisting of naftifine and haemoglobin were used for multimodal immunotherapy: the nanoparticles induced lipid peroxidation in *S. aureus* and sensitized it to host oxidant killing, while also recruiting neutrophils to eliminate it (Zhu et al. 2024).

Overall, combining multiple strategies or drugs within the same nanomedicine paves the way for synergistic effects. These are generally more efficient in eradicating infections, while also limiting the chances of generating resistance as evidenced by current medicines that address different targets of the human immunodeficiency virus (Temereanca and Ruta 2023).

#### Stimuli-responsive nanoparticles

As the distribution of drugs through the body can cause unwanted side effects at off-target sites, several stimuli-responsive nanoparticles have been designed that are activated and/or release their drug payload only in response to target-specific factors, such as pH or the presence of bacterial toxins. For instance, bacterial membrane-coated mesoporous SiO_2_ nanoparticles loaded with vancomycin were developed to release their load in an acidic environment and were able to kill MRSA in infected macrophages (Xie et al. 2023). Similarly, *S. aureus* can produce lipases that block the host immune system. Thus, drug carriers containing glycerolipids that are cleaved by these lipases can be used to release the antimicrobial cargo in a targeted way only at the infection site, where lipases are present. A similar approach was employed using phospholipid liposomes that were targeted to the α-hemolysin, which is a pore-forming toxin, and triggered the release of vancomycin *in vitro* (Pornpattananangkul et al. 2011). Although less specific, pH can also be used as a trigger for drug release. There is no “standard” formulation that works here as the pH can vary depending on the infection site, the bacterial strain, and the host inflammatory response. *Staphylococcus aureus* usually tends to infect wounds at higher pHs of about 7.5–8 and, once the infection is established, it can release components into the surroundings that decrease it. In other cases, such as lung and urinary tract infections, instead, *S. aureus* relies on the immune system to decrease the pH (Torres et al. 2017). Others have synthesized liposomes that are triggered by a low pH (6) causing structural changes that result in the release of encapsulated vancomycin at the target site.

Similar stimuli-responsive materials can help to improve the targeted delivery of antimicrobials exclusively at sites of infection, thereby increasing the efficacy while also limiting the occurrence of resistance at off-target sites.

#### Antivirulence strategies

Antivirulence therapies act on the virulence factors that are essential for bacterial colonization and infection. As these do not actively kill the bacteria, it is proposed that they are less likely to lead to resistance development and should allow the immune system to clear the bacterial infection. Such therapies have also been used in combination with other drugs for synergistic effects. Different small molecule inhibitors, antibodies, and polymers that have shown success against virulence factors released by pathogenic bacteria have so far been identified (Beckham and Roe 2014, Maura et al. 2016). In case of *S. aureus* infections, the major disadvantage is that these antivirulence strategies are intrinsically highly specific, whereas *S. aureus* produces a large number of toxins and other virulence factors (Sibbald et al. 2006, Ziebandt et al. 2010). Thus, designing molecules that are effective against all of them is quite challenging. One way to block virulence factors is to use plasma membrane-coated nanoparticles as nanosponges, which act as a decoy target by absorbing damaging toxins released by *S. aureus* on their surface, thereby diverting them away from their cellular targets (Hu et al. 2013b). These nanosponges have been used in mice to reduce the toxicity of α-hemolysin. They were further utilized for local treatment by immobilizing them onto a hydrogel, where they neutralized toxin at the injection site (Wang et al. 2015, Gao et al. 2016). Furthermore, Meng et al. (2009) created novel anionic liposomes loaded with anti-*mecA* phosphorothioate oligodeoxynucleotide and polyethylenimine to target the *mecA* gene, which restored the susceptibility of MRSA to β-lactam antibiotics. *In vitro*, the anti-*mecA* complex decreased *mecA* expression and inhibited MRSA growth.

Another successful approach of combinatorial nanoparticles involved coating polymer cores with red blood cell membranes. The membrane absorbed the toxins, while the core released antibiotics in response to enzymes produced by virulent bacteria. Neutralizing the toxin also allowed the immune system to more effectively clear out the infection (Li et al. 2014, Zhang et al. 2017).

#### Vaccines

The success of nanosponges in sequestering toxins sparked interest in their use for the purpose of vaccinations (Hu et al. 2013a, Wei et al. 2017). Bacterial toxins have been investigated for their potential use in vaccines, particularly α-haemolysin of *S. aureus*, but conventional techniques require protein denaturation to reduce their virulence, which can affect their efficacy in vaccination. Nanotoxoid vaccines were developed by simply mixing nanosponges with toxins, whereby they interact with surface receptors, while keeping their structure preserved without denaturation. These nanotoxoid vaccines did not show any toxicity when tested in mice, while successfully eliciting a higher immune response. This approach allows for the sequestration of a variety of toxins on the nanotoxoid surface, without necessarily knowing in advance their exact composition, thus providing a safe way of producing potentially multiantigenic vaccines. Taking this a step further, Wei et al. (2017) attempted coating gold nanoparticles with bacterial membranes directly, preserving the native pathogen associated molecular patterns and very diverse antigens on the nanoparticle surface. This resulted in higher antibody titers in vaccinated mice, and a three-fold better treatment when compared to the antigen not loaded on the nanotoxoid, demonstrating the potential of this approach.

### Reaching the pathogen

**Figure 7.**
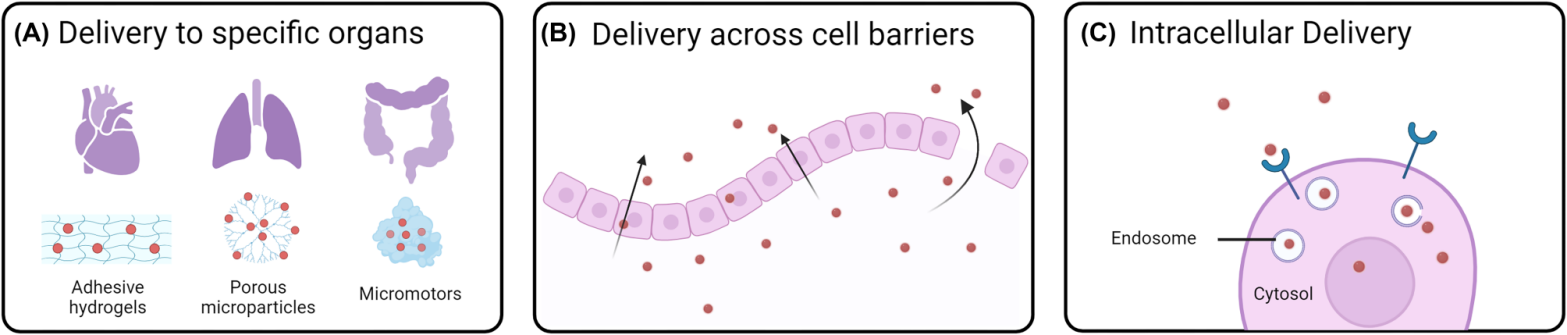
Schematic illustration of different barriers that nanoparticles have to overcome inside the human body for treatment of *S. aureus* infections. This includes the delivery of nanoparticles to the heart, lungs and gut with, for instance, adhesive hydrogels, porous microparticles and micromotors (A). Additionally, nanoparticles need to pass various cellular barriers (B) to reach sites of *S. aureus* infections, which may include intracellular compartments (C). Created with BioRender.com.

Once inside the body, pathogens have to go through several barriers at different anatomical organizational levels. These barriers also have to be crossed by any drug employed to treat these infections. Also in this regard, the unique properties of nanomaterials can be employed to circumvent some of these challenges, as described below for organs, tissues, and cells (Fig. 7).

#### Organ level

Certain organs and organ areas are naturally more challenging for drugs to reach because of physical limitations. Hybrid design is the key here, as it allows engineering flexibility in order to deal with the specific physical barriers encountered at these sites. The highly tunable nature of nanoparticles with respect to drug release and encapsulation allows additional design flexibility. This consideration applies specifically to areas that are subject to high flow conditions, where there is a very low retention time of the drugs, resulting in fast clearance. As an example of this, a high flow causing high shear stress is present in the endocardium of the heart, in the lumen of the urinary tract, in the gut lumen, and in lung airways. These are all sites that may present *S. aureus* infection, though some like urinary tract are less common (Raineri et al. 2021, Schuler et al. 2021).

Integrating nanoparticles into other materials can help deal with high flow conditions. Such conditions in the lung can be dealt with by combining nanoparticles with degradable porous micro particles that have a large size despite their light weight, allowing them to penetrate deep into the lung. Previous work showed that drug-loaded nanoparticles can be incorporated into the pores (Edwards et al. 1997, Crowder et al. 2002, Ohashi et al. 2009). These particles are inhaled dry and, upon encountering the moist environment inside the lung, the matrix dissolves, releasing the nanoparticles into the lung. This approach showed potential for the treatment of deep-lung infections, like tuberculosis, and they can be readily extended to MRSA.

Areas like the endocardium of the heart or the lumen of the urinary tract are subject to high flow and shear stress, which leads to very fast clearance of drugs. Nanoparticles can be combined with adhesive hydrogels in these areas to improve the retention time and, in this way, they allow sufficient time for the drug to show its effect locally. Kastrup et al. used an adhesive based on a catechol moiety, employed by mussels, to attach nanoparticles to various surfaces inside blood vessels in order to treat atherosclerotic plaques (Lee et al. 2007, Kastrup et al. 2012). This approach can, in principle, also be employed for other diseases like endocarditis and urinary tract infections. In contrast, Li and colleagues showed the use of micromotors that are capable of penetrating the mucus layer of the stomach, increasing the retention time of drugs in the stomach and gastrointestinal tract (Li et al. 2016a, 2017). These micromotors make use of water or stomach acid as fuel to help propel them for active drug delivery. They can autonomously neutralize gastric acid, rapidly depleting protons and restoring normal stomach pH within 24 h. They safely release a payload without causing acute toxicity or affecting stomach function.

To treat topical infections, Ingebrightsen et al. used a liposome-in-hydrogel system for the dermal delivery of chloramphenicol, which showed sustained drug release and limited skin permeation when tested *ex vivo* (Ingebrigtsen et al. 2017). As another example, collagen mimetic peptide-tethered vancomycin-loaded liposomes were encapsulated in collagen-based hydrogels for the treatment of local MRSA infections, and enhanced *in vitro* and *in vivo* antibacterial effects were demonstrated. MRSA infected wounds were successfully treated for up to 9 days with these liposomes when compared to the controls, even upon reinoculation of bacteria (Thapa et al. 2020).

#### Tissue level

Common issues encountered with conventional drugs relate to the way they move and distribute within the body which, in many cases, is primarily driven by diffusion and depends on their solubility in hydrophilic and hydrophobic environments (Nix et al. 1991). This is not controlled at all, and the indiscriminate diffusion results in off-target drug loss, and a limited dose actually reaching the target site, reducing the efficacy of drug-pathogen localization. Once at the target site, the antibiotics have to actually encounter the pathogen, with the additional factors of bacterial metabolism and excretion to consider.

Active drug targeting is an approach used for site-specific drug delivery. Ligands like aptamers (Schuler et al. 2021), antibodies (Bispo et al. 2020), peptides (Huang et al. 2009), and small molecules (Choi et al. 2012) are used to target bacteria or certain tissues. However, in the case of intracellular bacteria, it is quite difficult to distinguish an infected cell from a noninfected one from the outside, particularly when the bacteria have reached a state of persistence. Studies are ongoing to identify potential targets that are specific for infected cells and membrane proteomics can be used for their identification. Still, even when differentially expressed receptors are identified, designing targeted drugs remains a big challenge in drug delivery. To overcome the challenges associated with targeting, ligand purification and identification, in some studies nanoparticles were coated with the plasma membrane of cells from erythrocytes (Hu et al. 2011) and platelets (Hu et al. 2015) in order to allow for a biomimetic targeting approach (see also above). This actually reduced clearance of the nanoparticles by the immune system to a great extent. Similar approaches have the added advantage of allowing the core and membrane to be synthesized separately, providing flexibility in the engineering and design of both components. This strategy has been employed in cancer cell targeting as well (Gao and Zhang 2015).

Active targeting can also be achieved in response to certain compounds released by the bacteria or at the site of infection. Nanoparticles have been designed that remain inactive initially, with the drug release being triggered by the production of bacterial components. Examples of this are nanogels that degrade in response to active phosphatase or phospholipase produced by MRSA (Meng-Hua Xiong et al. 2012), and particles that transform in response to β-lactamase and other enzymes that commonly degrade antibiotic molecules (Li et al. 2016b). This resulted in improved wound healing in a mouse infection model. In addition to nanoparticle functionalization to achieve targeting, ligands can also be used to promote an immune response. For instance, liposomes encapsulating clarithromycin were modified with wheat germ agglutinin (WGA), and effective accumulation of liposomes was displayed in the enterocoelia of mice because of WGA. The number of MRSA colony-forming units in the kidney and spleen in mice treated with WGA-modified liposomal clarithromycin was significantly lower than that treated with free and nonmodified clarithromycin (Meng et al. 2016). Surface modification can also be used to prolong further the nanoparticle circulation time by preventing opsonization, the classic example being the addition of polyethylene glycol (Joralemon et al. 2010). A prolonged circulation time improves delivery to the target. For instance, in an *in vivo* study in a murine infection model, PEGylated vancomycin liposomes significantly prolonged the blood circulation time of the drug and, thanks to this, these liposomes increased the drug deposition in lungs, liver, and spleen, while reducing the drug concentration in kidneys and thereby its nephrotoxicity.

Bacteria can survive in more naturally acidic environments in areas like the stomach, vagina, and skin, or they can cause their surrounding environment to become acidic through their metabolic activity. In turn, this can lead to a reduction in drug activity allowing the bacteria to overcome antimicrobial therapy. Charge-responsive nanoparticles with a negative charge at physiological pH and a positive charge in more acidic environments, can be used to target the negatively charged bacterial cell wall under such conditions (Liu et al. 2009, Radovic-Moreno et al. 2012). Drug carriers have been designed that shield nontarget interactions at physiological pH, but bind to bacteria in acidity, delivering drugs and in part, mitigating the loss of drug activity with decreasing pH (Yu et al. 2018). These environment-specific nanoparticles are a necessary improvement over cationic nanoparticles which, as mentioned above, can be effective against the bacteria, but may also damage the negatively charged membranes of the host.

#### Cell level

Several bacterial pathogens have an intracellular lifestyle, either obligate like Listeria (Witter et al. 2016), Shigella (Mellouk and Enninga 2016), and Legionella (Hartland 2017) or facultative like *S. aureus*. As mentioned above, *S. aureus* bacteria are known to survive both inside immune cells, like macrophages, avoiding immune clearance and using them as a means to disseminate (Raineri et al. 2021), as well as in a variety of NPPCs, leading to chronic and recurrent infections (Baxt et al. 2013, Eisenreich et al. 2013). One of the problems in treating intracellular infections is that several of the current antibiotic classes, such as aminoglycosides and β-lactams show limited penetration into host cells, while others, such as fluoroquinolones and macrolides can penetrate, but remain inefficient as they cannot be retained inside the cell. The short half-life of these drugs requires a longer treatment time and large frequent doses, both of which can lead to off-target side effects. This increases treatment cost, lowers patient compliance and, overall, it contributes to the selection of antibiotic-resistant bacteria.

Within this context, nanomedicine provides excellent opportunities, especially where nano-sized drug carriers can be used to target intracellular *S. aureus* owing to their unique capacity to interact with and be internalized by cells. Active uptake allows the delivery of high doses of antibiotics inside cells. The ideal carrier should not only enable uptake of the drug into cells, but also allow the drug to be retained intracellularly at therapeutic concentrations for sufficient time, while also showing low toxicity and site-specific sustained release. This would decrease side effects, and lower the required dose to eradicate the infection. Nanoparticles are the perfect vehicles to overcome the cellular barrier and deliver antibiotics inside cells, as they are actively endocytosed by cells, thus increasing the probability of the drug reaching its target pathogen inside the cell. They are also very easily taken up by immune cells like macrophages (Chellat et al. 2005, Edagwa et al. 2014), and the uptake by macrophages can be enhanced by use of targeting ligands such as mannose to target the mannose receptor, or maleylated bovine serum albumin to target scavenger receptors, among others. These approaches have been used with varying degrees of success against tuberculosis and MRSA (Vyas et al. 2004, Pumerantz et al. 2011, Maya et al. 2012, Sémiramoth et al. 2012, Lee et al. 2015, Kamaruzzaman et al. 2016). Another approach is to use anionic liposomes as carriers, which are more likely to be phagocytosed by macrophages as they resemble the negatively charged bacteria and can, thus, be used to target bacteria residing within these immune cells.

Once inside the cell, nanoparticles may or may not require endosomal escape, depending on where inside the cell the intracellular bacteria reside. In the case of intracellular infections where the bacteria remain confined in the endo-lysosomal compartment following internalization, nanoparticles are the ideal drug carriers, given that they are internalized by cells via endocytosis, thus reaching the same compartments. Instead, in the case of cytosolic bacteria or bacteria reaching other compartments outside the endo-lysosomal system, endosomal escape is necessary. In these cases, as also discussed above, polymers and lipids which respond to pH have been employed in order to achieve endosomal escape (Gehanno et al. 1996, Murthy et al. 2003, Mkandawire et al. 2009, Misra and Sahoo 2010). These materials can become positively charged once inside the endosomes, because of their slightly lower pH. Once positively charged, they can disrupt or destabilize the endosome and, in this way, release their cargo into the cytosol (Murthy et al. 2003). Some liposomes also have the capacity to fuse with the host cell membrane to release their cargo directly into the cytosol (Huang et al. 2011). In addition, specialized surface modifications on nanoparticles can impart unique properties. For instance, the ultrasmall amphiphilic gold nanoparticles developed by Stellacci (Verma et al. 2008) are capable of entering cells in an energy-independent manner, allowing them to diffuse into the entire volume of the cell. This type of nanoparticles can reach intracellular bacteria directly, without needing endosomal escape mechanisms and may be used to treat intracellular infections (Yedavally et al. 2025).

Another requirement for intracellular drug carriers is the ability to maintain sufficient levels of the drug inside the cell. Several strategies can be used to achieve this. For instance, PLGA nanoparticles allowed a sustained gentamicin release for 25 days, and isoniazid release for 14 days (Kohane et al. 2006, Lecaroz et al. 2006).

In conclusion, nanoparticles offer multiple opportunities for the treatment of intracellular infections. Different strategies could be used to target nanoparticles to specific organs and cell types that are known to be infected by bacteria. Distinguishing within these organs the infected cells where the bacteria persist, in order to deliver their payload specifically in these cells, may further enhance efficacy.

#### Biofilms

Alongside intracellular infections, biofilms present another vexing challenge for antimicrobial therapies. The biofilm matrix may sequester bacterial cells from the exterior environment (Götz 2002). Efflux pumps play a role, affecting the exclusion and inclusion of quorum-sensing biomolecules and waste and byproducts responsible for biofilm formation. The matrix of ‘‘extracellular polymeric substance’’ (EPS) and extracellular DNA present imparts an overall negative charge, and can cause antibiotics like aminoglycosides to stick, thereby immobilizing them on the biofilm surface. Consequently, antibiotics exert their effects only on the upper layers of a biofilm. Furthermore, the altered microenvironment inside a biofilm, such as low pH and low oxygen availability, can compromise the efficacy of the antimicrobials, and the biofilm may even contain enzymes that can destroy them directly. Importantly, as pointed out above, bacteria embedded within a biofilm show slow growth, if any, and have downregulated their metabolism.

**Figure 8.**
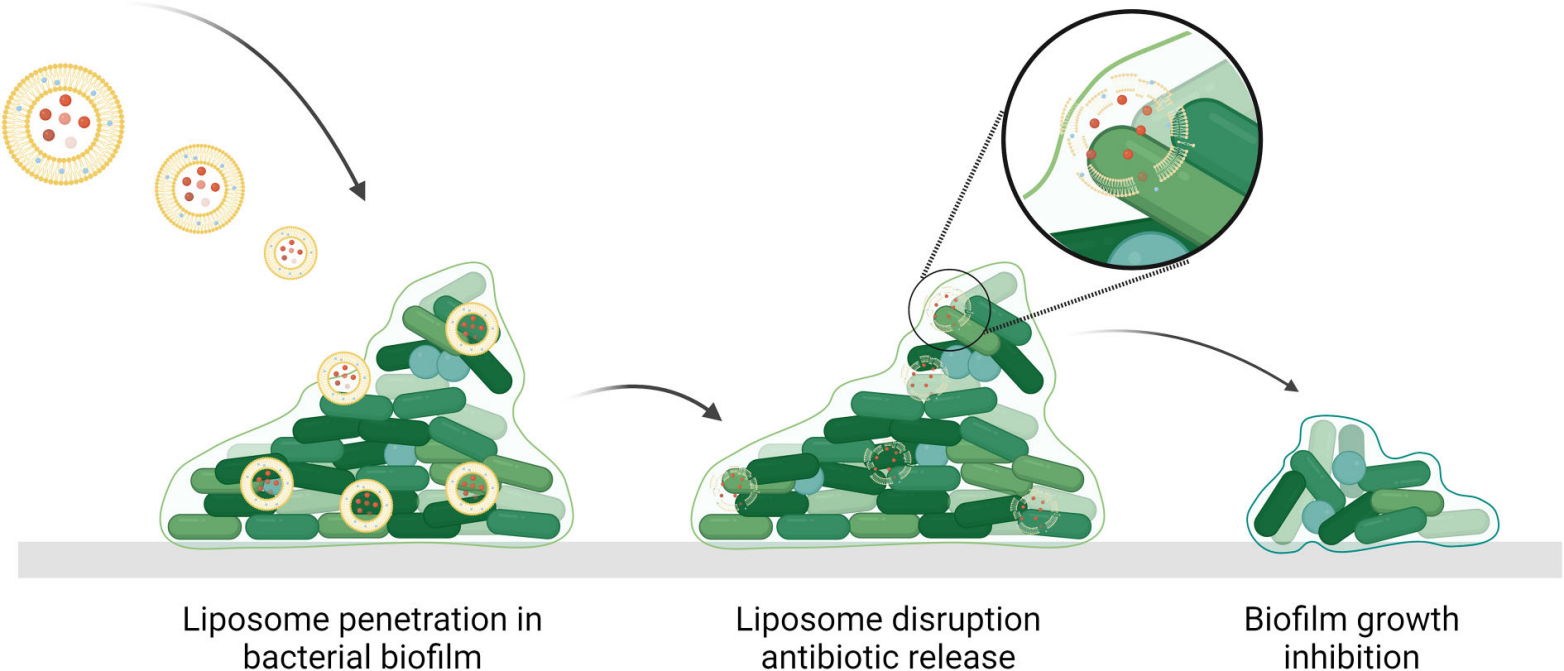
Liposome penetration in biofilms: biofilms are considered a particular challenge due to the lower penetrability of the antibiotic in their extracellular matrix. Antibiotic-loaded liposomes can interact with bacteria organized in a biofilm, enabling antibiotic delivery within the biofilm structure. Figure adapted from Ferreira et al. (2021). Created with BioRender.com.

Several nanoparticle-based solutions outlined in preceding sections can be employed also against biofilms. For instance, blocking efflux pumps as detailed earlier can result in preventing the export of waste products or impairing quorum cell-cell signaling, both of which have a detrimental effect on biofilms. The altered microenvironment can be a means of targeted drug release, for instance pH or enzyme-triggered drug release (Wu et al. 2020). Combinatorial use of metal nanoparticles along with conventional antibiotics can greatly enhance biofilm inhibition when compared to the individual components. Selenium nanoparticles combined with ampicillin, oxacillin and penicillin showed strong effects against biofilms (Cihalova et al. 2015). Another example of combinatorial nanoparticles comes from wheat germ agglutinin-modified liposomes encapsulating clarithromycin, which inhibited the formation of biofilms and enabled the disassembly of pre-existing biofilms (Meng et al. 2016). More in general, antibiotic-loaded liposomes can interact with bacteria organized in a biofilm, enabling antibiotic delivery within the biofilm structure as depicted in Fig. 8. For instance, fusogenic vancomycin liposomes showed an enhanced effect against mature biofilms, and liposomes loaded with biosurfactants from *Lactobacillus* were considered as antibiofilm agents against MRSA biofilms (Giordani et al. 2019). These liposomes were noncytotoxic and prevented biofilm formation by MRSA isolates.

In addition to biofilm disruption, surface treatments using nanotechnology can also be employed to deal with biofilms in other multiple ways. Surfaces can be engineered to prevent adhesion or kill on contact, for instance using a sharp nanostructured surface, or by the release of antimicrobials from the surface, which can also be stimulus-responsive. Bacteria-targeted antimicrobial photodynamic therapy has also been used to great effect against biofilms both *in vitro* and *in vivo* (Gupta et al. 2017, Bispo et al. 2020, 2022b).

While several strategies can be employed to use nanoparticles against biofilms, access to the lower layers can constitute a barrier also for nanoparticle-based treatments depending on their capacity of penetration. In this regard, the larger size of nanoparticles in fact can constitute a challenge for biofilm penetration compared to what is possible with small molecule drugs. Interestingly, antibiotic-loaded small amphiphilic gold nanoparticles were shown to be able to penetrate biofilms throughout their thickness and induce death of biofilm-embedded bacteria (Yedavally et al. 2025).

## Further considerations

### Clinical development and challenges in the translation of antimicrobial nanomedicines

#### Clinical status of antimicrobial nanomedicines

As discussed in the previous sections, nanoparticles offer multiple opportunities for the treatment of infections by pathogens, such as *S. aureus*. However, when looking at their status in the clinical development, it is clear that most of the described applications are still at the preclinical stage, while the clinical application of antimicrobial nanoparticles is still in its infancy. A systematic search of ClinicalTrials.gov, with a cut-off date of 7 July 2025 identified 61 registered clinical studies employing nanomaterials for the prevention, diagnosis, or treatment of infectious diseases. An overview of this analysis is given in Table 1.

**Table 1.** **Statistics on the clinical status of antimicrobial nanomedicines**.

Trial status	Nanoparticles/nanomedicine	Bacterial infections/AMR	*Staphylococcus aureus*	*Overlap (nanoparticles/nanomedicines against bacterial infections)*
Active, recruiting	223	2770	68	*8*
Completed	339	8540	295	*35*
Withdrawn/suspended/terminated/unknown	231	3648	129	*18*
	793	11 160	492	*61*

The table summarizes the results of a systematic search of ClinicalTrials.gov, with a cut-off date of 7 July 2025. The numbers of clinical trials involving nanoparticles or nanomedicines, as well those related to the treatment of bacterial infections and, within these, the trials specifically focused on *S. aureus* are given together with the trial status. Finally, the number of clinical trials with nanoparticles or nanomedicines against infectious diseases is also included.

The current clinical portfolio is largely exploratory, with 35 studies reaching primary completion and the remainder distributed among active recruitment, prerecruitment registration, and indeterminate, terminated, or withdrawn status. Study designs are predominantly single-center, with a median sample size of 50 participants.

More than 60% of the identified trials focus on dental and periodontal diseases, leveraging the advantages of topical administration, reduced systemic exposure, and quantifiable outcomes such as International Caries Detection and Assessment System (ICDAS) scores or salivary bacterial load. For instance, ongoing studies are assessing a fluorescent starch nanoparticle rinse for early caries (NCT06761248) and a doxycycline-loaded PLGA gel in periodontitis (NCT06994455). A smaller subset investigates wound and soft tissue infections. The most substantial ongoing study randomizes 170 patients in a double-blind comparison of a silver-based dressing versus Aquacel Ag + for diabetic foot ulcers (NCT06667752). Additional studies address tuberculosis (e.g. nanodisk-MS antigen capture, NCT03271567), fungal dermatoses, parasitic skin diseases, keratitis, and sepsis or organ failure. Only one completed trial specifically targeted methicillin- and vancomycin-resistant *S. aureu*s using bactericidal silver nanoparticles (NCT04431440).

These studies reflect four principal technological approaches. First, metallic nanoparticles such as silver, zinc oxide, and titanium dioxide are incorporated into dressings, varnishes, orthodontic appliances, and restorative materials for their broad-spectrum antimicrobial activity. Second, polymeric and liposomal carriers including PLGA and chitosan matrices are used to deliver conventional antibiotics at high local concentrations while minimizing systemic exposure. An example is the doxycycline-PLGA gel for periodontitis currently in recruitment. Third, catalytic and biofilm-reactive formulations harness redox properties to generate ROS or disrupt extracellular matrices, and the evaluation of a ferumoxytol/H₂O₂ protocol for root-canal disinfection (NCT06110494) exemplifies this strategy. Finally, diagnostic platforms incorporate nanoparticles to improve detection sensitivity, as seen in trials of fluorescent starch rinses for early caries (NCT06761248), nanodisk-based mass spectrometry for *Mycobacterium tuberculosis* antigen capture (NCT03271567), and titanium-dioxide nanotube breath analysis for tuberculosis triage (NCT02681445). Notably, none of these diagnostic studies specifically detects *S. aureus*.

Some methodological limitations are apparent across the portfolio. Most trials are small and their monocentric nature restricts generalizability across different healthcare environments and microbial populations. Outcome measures are heterogeneous, ranging from ICDAS criteria in dental studies to reduction in wound area or time to closure in soft tissue infection trials. This variability complicates cross-study comparisons and meta-analysis. Furthermore, public reporting remains inconsistent, particularly regarding study outcomes and physiochemical characterization of nanomaterials such as size distribution, surface charge, drug loading, and release kinetics.

Despite the clinical importance of *S. aureus* in many targeted conditions, few studies incorporate quantitative, species-specific endpoints, such as culture, PCR, or biofilm biomass measurements. To date, nanoparticle-based formulations intended for systemic or inhalational administration have not reached human testing, reflecting concerns related to biodistribution and long-term safety. This limitation constrains translation toward the treatment of systemic infections, including bacteremia, pneumonia, or prosthetic-joint infections.

In summary, current clinical investigation of antimicrobial nanoparticles is concentrated in localized, early-phase studies addressing dental and skin infections, with metallic and polymeric formulations prevailing and diagnostic applications emerging. However, evidence for efficacy against *S. aureus* remains limited and development of systemic therapies has yet to progress to human trials. Future advancements will require larger, multicenter studies with harmonized, organism-specific endpoints and transparent reporting of both clinical outcomes and nanoparticle properties. Progress in these areas will be essential for bridging the gap between promising laboratory findings and clinical application. Until then, the promise of antimicrobial nanoparticles against staphylococcal disease remains a compelling prospect rather than an established clinical reality.

#### Challenges in the clinical translation of antimicrobial nanomedicines

Several aspects that limit the translation of antimicrobial nanomedicines and that explain their current (non-)clinical status, are common to all nanomedicine applications. Reviews on this topic have summarized and critically analyzed the challenges in the clinical development of nanomedicines in details, while also suggesting strategies to address them and speed up the approval of novel nanomedicine products (Hua et al. 2018, Metselaar and Lammers 2020, Younis et al. 2022). For instance, aspects limiting nanomedicine clinical translation include the need of demonstrating efficacy in comparison to available treatments, as well as the need of demonstrating safety. In relation to safety, some aspects on the potential toxicity of nanomedicines and their fate within the body still need to be elucidated. Organic nanoparticles composed of polymers and lipids are usually biodegradable, and are therefore the currently most utilized carriers. On the other hand, inorganic materials, which include many of the examples discussed for antimicrobial applications, including a large portion of those currently in clinical trial, can accumulate inside tissue, particularly the liver and spleen. Small nanoparticles (<5 nm) can be cleared through the kidneys, bladder, and urine, but larger ones need careful consideration before their use. Long-term toxicity studies need to be performed to evaluate whether they may induce undesired effects at the sites where they may accumulate. While several studies assessing nanoparticle safety seem to exclude toxicity in many cases, most of the studies have been done *in vitro* or in animal models, especially murine models. While these do provide an indication concerning the efficacy of drug delivery, the results do not directly translate to humans. In order to bridge this gap and demonstrate safety as well as efficacy in humans, the field has recognized the need to develop better testing models using human materials, such as *ex vivo* tissue slices or three-dimensional organoids and organs-on-chips. Similarly, while many studies have demonstrated promising nanoparticle effects on bacteria in isolation, further testing needs to be done to ensure that they are efficient also in more complex models, while also excluding additional unwanted side effects on the host.

### Generation of resistance against antimicrobial nanotechnologies

**Figure 9.**
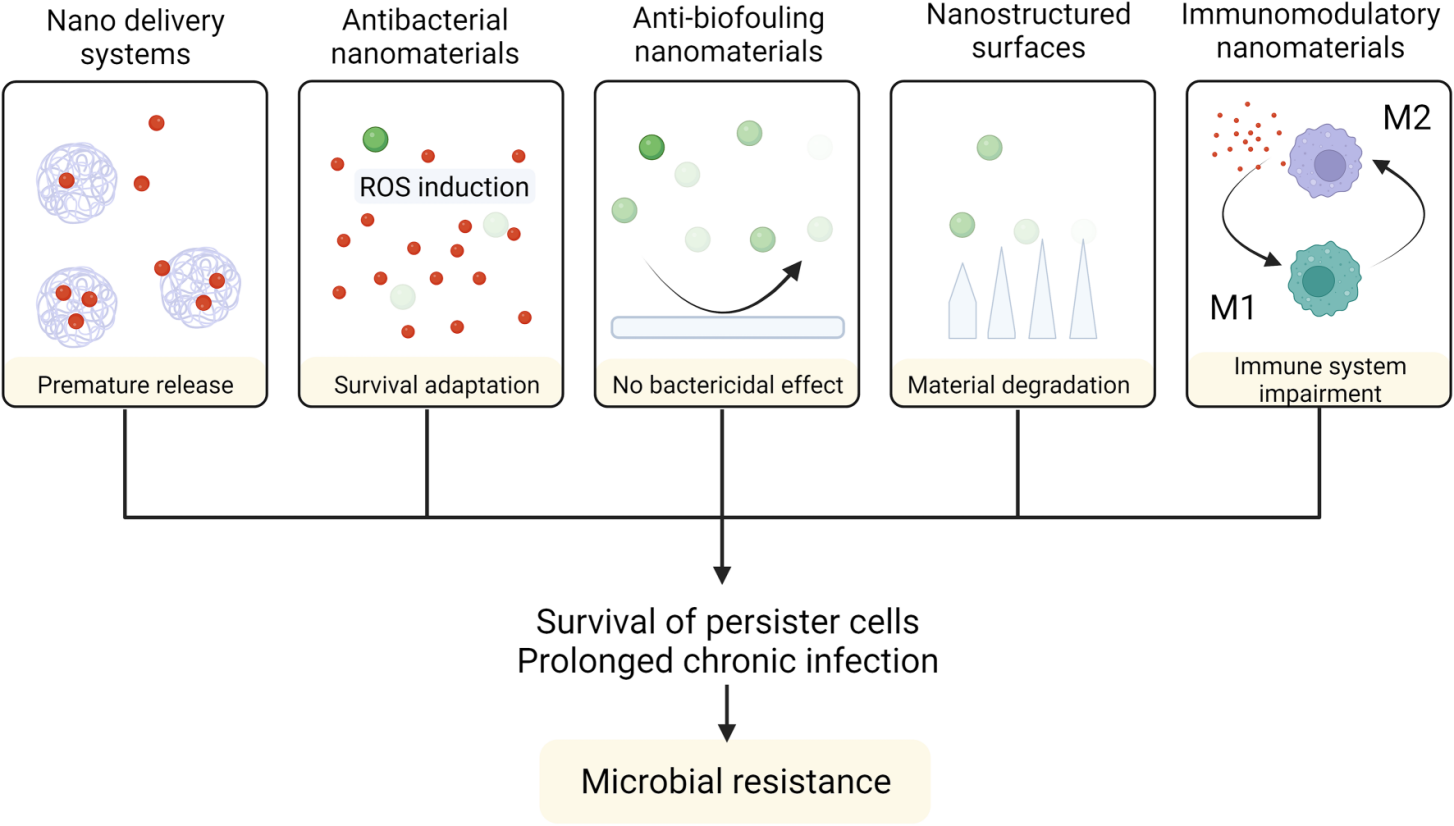
Different mechanisms that may lead to bacterial resistance against antimicrobial nanotechnologies. Figure adapted from Wu et al. (2022). Created with BioRender.com.

Another important question that needs to be addressed is whether microbial resistance can develop even in response to the new nanotechnologies and some of the discussed mechanisms of action of nanomaterials. Fig. 9 outlines some examples how different nano-based systems could conceivably lead to bacterial resistance.

For instance, liposomes are prone to premature drug release. Thus, further optimization is necessary to avoid similar effects, which would limit their efficacy. The use of stimuli-responsive nanoparticles could be a potential solution in order to achieve drug release only at the infection site. While it seems still rare so far, resistance to metal nanoparticles has already been reported. In particular, some bacteria were observed to release filaments that can capture metal ions, thereby reducing the efficacy of mechanisms relying on the release of ions by metal nanoparticles (Graves et al. 2015). Antibiofouling materials that prevent adhesion of bacteria have shown effect in preventing biofilms, but they do not actually kill the bacteria, which can still circulate in a planktonic or intracellular form. Nanostructures meant to puncture bacterial membranes may degrade over time and, hence, they may need to be replaced to ensure that they continue working. Finally, several nanoparticles target immune cells, but they could have the undesired effect of weakening the immune defenses, or diverting the immune system from clearing the infection. More careful design needs to be applied to minimize such immune system impairment. For example, stealth coatings and PEG do help to avoid unwanted interactions with the immune system, and further techniques like plasma membrane coating are also being studied (Jiménez-Jiménez et al. 2020).

Overall, the preferred solution to avoid or minimize potential resistances would be to use combinatorial nanoparticles that rely on more than one antimicrobial mechanism, or nanoparticles that rely on physical disruption of the bacteria, which seem less likely to be overcome.

## Conclusion

*Staphylococcus aureus* is a very dangerous versatile pathogen with a great potential for developing antimicrobial resistances. New methods to treat *S. aureus* infections will therefore be needed continuously. Nanomedicine offers multiple attractive opportunities to develop new treatments against *S. aureus* infections, that take advantage of the small size and unique properties of nano-sized materials. Nanoparticles can be used as drug carriers to improve the delivery of antimicrobials, but in some cases, they possess intrinsic antimicrobial properties that can also be exploited. Added to this, they are highly tunable and offer extensive engineering flexibility, thereby allowing the combination of multiple therapies at once. Thus, nanoparticles can be used to help in dealing with *S. aureus* resistance to antimicrobials, the treatment of MRSA, as well as with reaching the pathogen in difficult-to-reach environments, like biofilms and intracellular compartments where the bacteria may persist. Many studies have already showcased such potential applications of nanoparticles in the fight against staphylococcal infections. However, in order to bring nanomedicine-based antimicrobials to the clinic, more studies still need to be done to test their efficacy in more complex infection models, including humanized models. Importantly, potential long-term effects induced by these materials still need to be excluded, and it will be necessary to determine their clearance from the body of the host. It will also be relevant to test whether they may elicit resistance in *S. aureus* and other targeted pathogens. With careful consideration of these aspects, the many opportunities offered by nanomedicine and, in particular, the combinatorial therapies enabled by these advanced technologies are likely to find successful applications in the fight against *S. aureus*.
